# Synergy of Ascr#11 and Improved Aeration Drives Enhanced Yield and Fitness of Entomopathogenic Nematodes

**DOI:** 10.3390/life16050703

**Published:** 2026-04-22

**Authors:** Qiji Wang, Huilin Liao, Dzmitry Voitka, Alena Yankouskaya, Richou Han, Yongling Jin, Li Cao

**Affiliations:** 1 College of Agriculture, Heilongjiang Bayi Agricultural University, Daqing 163319, China; 2 Guangdong Key Laboratory of Animal Conservation and Resource Utilization, Guangdong Public Laboratory of Wild Animal Conservation and Utilization, Institute of Zoology, Guangdong Academy of Sciences, Guangzhou 510260, China; 3 Institute of Plant Protection, National Academy of Sciences of Belarus, Mira Str. 2, Ag. Priluki, Minsk District, 223011 Minsk, Minsk Region, Belarus

**Keywords:** ascarosides, biocontrol agents, in vitro production, liquid culture, targeted metabolomics

## Abstract

Entomopathogenic nematodes (EPNs) are crucial biocontrol agents, yet optimizing the yield and quality of infective juveniles (IJs) during commercial liquid production remains challenging. This study utilized a central composite rotatable design to optimize liquid culture parameters (ascaroside, dimethyl sulfoxide, medium volume, IJ inocula) for *Heterorhabditis bacteriophora* H06 and *Steinernema carpocapsae* All. The results demonstrated that improving aeration (inferred from reduced media volume), combined with ascr#11 regulation, synergistically enhanced IJ yield and quality. Under optimized conditions, yields reached 3.35 × 10^5^ IJs/mL for *H. bacteriophora* H06 and 2.67 × 10^5^ IJs/mL for *S. carpocapsae* All. Crucially, the IJs from the high-yield flask exhibited significantly superior infectivity (24–26% single-IJ infection rate) compared to solid-culture controls (13–14%). Targeted metabolomics profiling of sugar, energy and fatty acids of *H. bacteriophora* H06 revealed upregulated tricarboxylic acid (TCA) cycle intermediates (citrate, pyruvate) and the significant accumulation of stress-protectant trehalose and immune-modulating polyunsaturated fatty acids (eicosapentaenoic acid, arachidonic acid). These findings establish a fermentation strategy that simultaneously enhances IJ yield and biological quality by reducing media volume (used as a proxy for improved aeration) and supplementing ascr#11. Furthermore, the distinct metabolic profile enriched in energy, stress, and immune-modulating metabolites identified in *H. bacteriophora* provides a plausible explanatory framework for the parallel phenotypic improvements observed across both species.

## 1. Introduction

The global agricultural sector faces growing pressure to reduce reliance on broad-spectrum synthetic pesticides, driven by arthropod resistance, environmental contamination, and stringent regulations. Entomopathogenic nematodes (EPNs) of the genera *Steinernema* and *Heterorhabditis* have emerged as prominent biological control agents [1,2]. These obligate parasites, functioning in symbiosis with bacteria (*Xenorhabdus* spp. and *Photorhabdus* spp.), possess the unique capacity to actively seek hosts within the soil matrix, making them exceptionally effective against cryptic pests that escape conventional surface treatments [3].

However, transitioning EPNs from niche biocontrol agents to global agricultural staples depends on scalable, cost-effective production [4]. While in vivo production yields nematodes of high biological quality, it is labor-intensive and difficult to scale [5]. Consequently, in vitro liquid fermentation remains the viable pathway for mass production [6,7,8]. Yet, this method is historically plagued by a persistent limitation: the inverse relationship between production intensity and biological quality, a phenomenon widely recognized in EPN mass production [8,9]. This phenomenon implies that optimizing for maximum biomass in bioreactors frequently results in the IJs with compromised physiological traits [10]. These liquid-cultured nematodes often display reduced longevity, lower lipid reserves, and diminished pathogenicity compared to those reared in their natural insect hosts (in vivo) or on solid media [11,12,13]. This degradation is widely attributed to the artificial nature of the liquid environment, which lacks the intricate cues of the insect hemocoel and imposes stressors such as hydrodynamic shear and hypoxia [7,14].

Biologically, the IJ is a non-feeding, developmentally arrested stage analogous to the *Caenorhabditis elegans* (Maupas) dauer larva. Its survival and infectivity rely entirely on energy reserves (lipids and carbohydrates) accumulated during preceding developmental stages. In high-density fermentation, insufficient oxygen mass transfer efficiency (OTR) relative to metabolic demand can prematurely trigger dauer formation before these reserves are replete, resulting in nutrient-depleted nematodes with poor shelf-life [7,15]. Physiologically, the synthesis of these fitness-related reserves relies heavily on aerobic metabolism. For instance, the biosynthesis of polyunsaturated fatty acids requires molecular oxygen as an electron acceptor for Δ 5-desaturase [16]. Similarly, the transport of succinate into mitochondria by uncoupling protein 4 (UCP4) is a rate-limiting step for Complex II-mediated respiration [17]. In high-density liquid fermentation, oxygen limitation suppresses these pathways, diverting metabolic flux towards anaerobic glycolysis and preventing the accumulation of functional metabolites [10,15]. Therefore, the challenge lies not merely in increasing numbers, but in engineering a fermentation environment that mimics the host’s nutritional and signaling landscape.

To bridge the gap between artificial fermentation and host ecology, recent research has investigated nematode chemical communication. Ascarosides, a conserved family of glycolipids, serve as the primary mode of chemical communication in entomopathogenic nematodes [18,19,20,21,22,23,24,25,26,27]. These small molecules act as potent pheromones regulating density-dependent behaviors, including aggregation and entry into the dauer stage [22,25,28,29]. We hypothesized that the exogenous addition of specific ascarosides—specifically ascr#11—could serve as a synchronization signal in liquid culture. By chemically simulating “host depletion” at the precise moment of peak vegetative biomass, we aimed to coordinate a uniform population exit into the IJ stage, thereby minimizing energy wastage on futile reproductive cycles.

Alongside signaling molecules, chemical chaperones such as dimethyl sulfoxide (DMSO) have been explored to enhance fermentation performance. Previous studies in our laboratory indicated that low concentrations of DMSO can significantly improve EPN yield [26,30], potentially via a hormetic stress response. Moreover, DMSO has been shown to exert complex influences on cellular physiology and secondary metabolism [31,32]. Building on these previous findings, this study aimed to identify an optimized multifactorial combination—including ascr#11, DMSO, and oxygenation levels—to maximize both the quantity and quality of EPNs in liquid culture. To achieve this, *Heterorhabditis bacteriophora* (Poinar) and *Steinernema carpocapsae* (Weiser) were strategically selected because they represent the two major entomopathogenic nematode families (Heterorhabditidae and Steinernematidae) widely utilized in biocontrol of important pests [1,8], with fundamentally distinct reproductive strategies (hermaphroditic vs. amphimictic). We hypothesized that if the synergistic application of ascr#11 and improved aeration could concurrently enhance both yield and biological fitness in both species, it would indicate the broader applicability of this fermentation strategy across different EPN families.

Finally, while yield is a quantitative metric, “quality” is a complex trait involving infectivity and storage stability [8,33,34]. Traditional bioassays provide a functional endpoint but fail to capture the underlying physiological state. Targeted metabolomics offers a powerful tool to dissect the molecular basis of quality. Given the non-feeding nature of IJs, we specifically targeted metabolites governing three core fitness traits: sugars such as trehalose for environmental stress tolerance [33], tricarboxylic acid (TCA) cycle intermediates for motility-driven energy flux [35], and polyunsaturated fatty acids (PUFAs) for immune modulation potential [36]. By quantifying these key biomarkers, we can construct a “metabolic profile” of the optimal IJ [11,37].

This study utilizes a central composite rotatable design (CCRD) to systematically evaluate the synergistic effects of ascr#11, DMSO, media volume (oxygenation), and IJ inoculum size on the liquid culture of *H. bacteriophora* H06 and *S. carpocapsae* All. The specific objectives are: (1) To define the parameters that maximize yield while maintaining high biological quality; (2) To elucidate the hierarchical relationship between physical oxygenation and chemical signaling; and (3) To characterize the metabolic profile associated with the “high-yield and high-quality” phenotype, thereby establishing new biomarkers for fermentation quality control.

## 2. Materials and Methods

### 2.1. Nematode and Bacteria

*Heterorhabditis bacteriophora* H06 and *Steinernema carpocapsae* All were obtained from the Centre for Bioresource and Bioengineering, Institute of Zoology, Guangdong Academy of Sciences (Guangzhou, China). Their specific symbiotic bacteria, *Photorhabdus luminescens* (Thomas et Poinar) (for *H. bacteriophora* H06) and *Xenorhabdus nematophila* (Poinar et Thomas) (for *S. carpocapsae* All), were originally isolated from the hemolymph of infected *Galleria mellonella* (Linnaeus) larvae. Bacterial stocks were maintained in 15% glycerol at −80 °C. Prior to use, bacteria were streaked onto NBTA plates (nutrient agar supplemented with 0.004% (*w*/*v*) triphenyl tetrazolium chloride and 0.0025% (*w*/*v*) bromothymol blue) to ensure the selection of primary phase variants, which are essential for supporting nematode development [38,39].

*G. mellonella* (greater wax moth) larvae were reared in the laboratory on a standard artificial diet consisting of wheat flour, yeast, honey, and glycerol [40]. Late-instar larvae were harvested for in vivo nematode propagation and infectivity assays. Although *G. mellonella* is an invertebrate species and therefore exempt from formal ethical approval by an Institutional Animal Care and Use Committee (IACUC), all procedures regarding the rearing, handling, and disposal of the insect larvae were conducted in strict accordance with general animal welfare principles and the ARRIVE guidelines where applicable, ensuring the minimization of any potential suffering.

### 2.2. Reagents and Medium Components

Ascaroside #11 (ascr#11) was synthesized by WuXi AppTec (Tianjin, China) with a purity > 95% and stored at −20 °C. Dimethyl Sulfoxide (DMSO, analytical grade) was purchased from Tianjin Fuyu Fine Chemical Co., Ltd. (Tianjin, China). Media components included: yeast extract and tryptone (Oxoid Ltd., Basingstoke, UK); agar (BioFroxx, Einhausen, Germany); nutrient broth (BD, Sparks, MD, USA); wheat flour (COFCO International, Beijing, China); soybean flour (Xinlong Asia Longkou Soybean Food Co., Ltd., Longkou, China); corn oil (Guangdong Hexing Edible Oil Co., Ltd., Guangzhou, China); and dried egg yolk (Jiangsu Kangde Egg Industry Co., Ltd., Nantong, China).

### 2.3. Preparation of Nematode Inoculum

To ensure physiological uniformity, IJs used for liquid fermentation inoculation were produced via solid sponge culture [13,14,41]. Briefly, autoclavable sponge pieces were impregnated with a lipid-rich medium, consisting of 15% wheat flour, 5% soybean flour, 5% corn oil, 1% yeast extract, and 1% dried egg yolk. These diet-impregnated sponges were inoculated with primary phase symbiotic bacteria, followed by introducing the IJs 2 days later. Cultures were incubated at 25 °C for 4 weeks. Emerging IJs were harvested and washed three times with sterile water by natural sedimentation. Because the IJs were produced in pre-established monoxenic sponge cultures, they were not chemically surface-sterilized prior to the liquid fermentation inoculation to avoid potential chemical stress. Instead, a sample of the washed IJ suspension was plated onto NBTA agar to verify the absence of any microbial contaminants. The verified monoxenic IJs were then stored at 15 °C on a shaker (120 rpm) for no more than 3 days prior to experimentation.

### 2.4. Liquid Culture Protocol

Monoxenic liquid cultures were conducted in 250 mL Erlenmeyer flasks. The basal medium, adapted from Han [14], consisted of 1.8% wheat flour, 3.2% soybean flour, 0.7% yeast extract, 0.4% tryptone, 1.1% dried egg yolk, 2.2% corn oil, 0.5% NaCl, and 90.2% distilled water. All components were homogenized and autoclaved at 121 °C for 40 min. Following cooling, flasks were inoculated with 2% of a 48 h symbiotic bacterial culture and incubated at 25 °C, 120 rpm for 3 days to establish the bacterial lawn. Subsequently, the IJs were inoculated, and ascr#11 (ranging from 0.0002 to 2 pM for *H. bacteriophora*, and 0.0002 to 2 nM for *S. carpocapsae*) and DMSO (ranging from 0.0025% to 0.04% *v*/*v*) were added via sterile filtration (0.22 µm), according to the experimental design (see Section 2.5). Flasks were incubated at 25 °C with agitation speed increased to 140 rpm for 28 days.

### 2.5. Experimental Design

To model the non-linear interactions between variables, a four-factor, five-level central composite rotatable design was employed. The independent variables and their corresponding levels are detailed in Table 1. Briefly, the factors investigated were: (Factor A) Ascaroside #11 concentration: Based on preliminary sensitivity assays [26], concentration ranges were species-specific: pM range for *H. bacteriophora* H06 and nM range for *S. carpocapsae* All. (Factor B) DMSO concentration (% *v*/*v*). (Factor C) Media volume (mL/flask): Used as a proxy for Oxygen Transfer Rate (OTR); lower volumes in a fixed-size flask result in higher surface-to-volume ratios and improved aeration. (Factor D) Inoculum size (IJs/mL) at five gradient levels of 1500 IJs/mL (coded value = −2), 3000 IJs/mL (coded value = −1), 6000 IJs/mL (coded value = 0), 12,000 IJs/mL (coded value = 1), and 24,000 IJs/mL (coded value = 2). The design matrix included 36 flasks (16 factorial points, 8 axial points, and 12 center points to estimate pure error) (Supplementary Table S1). Each run was performed with three independent biological replicates, where a single replicate was defined as one independent flask.

### 2.6. Quantification of Yield and Biological Quality

The yield of the IJs was counted using a counting slide under a stereomicroscope at day 28 after inoculation; for each flask, the yield was determined by averaging the IJs from three separate samples. Data are expressed as IJs/mL.

“One-on-one” bioassay was utilized [26,42,43] to rigorously assess individual nematode fitness. A single viable IJ was transferred in 20 µL water onto a sand column (30 g sand, 6% moisture) containing one *G. mellonella* larva. Infection was assessed after 96 h. Cadavers were dissected to confirm nematode infection. n = 60 insects were used per treatment (3 replicates of 20 insects).

IJ recovery was assessed by incubating approximately 100 IJs in cell culture wells containing cell-free symbiotic bacterial supernatant [27,44,45]. Recovery rate was calculated as the percentage of IJs that resumed development after 3 days and 6 days [45].

Dispersal behavior of the IJs was measured on 2% agar plates. Approximately 100 IJs were placed in a central circle (1.1 cm diameter). The proportion of nematodes migrating out of the circle was recorded after 5 min for *H. bacteriophora* H06 or 20 min for *S. carpocapsae* All, according to the methods described by Kaplan et al. [22] and Wang et al. [27]. All subsequent biological quality assessments (infectivity, recovery, and dispersal) were performed using three biological replicates (three separate flasks), with each phenotypic assay repeated in triplicate for each flask.

### 2.7. Targeted Metabolomics

Targeted metabolic profiling was performed by Metware Biotechnology Co., Ltd. (Wuhan, China). *H. bacteriophora* H06 was selected as the model organism due to its hermaphroditic reproduction [46], which ensures developmental synchrony in liquid culture. IJs were collected from the optimized “High Yield” group (No. 21), the “Low Yield” group (No. 23), and the Solid Culture Control (CK). Six biological replicates were prepared per group.

For sample preparation, approximately 70,000–100,000 IJs were collected per sample. After washing and centrifugation (10,000 rpm, 10 min), the IJ samples were snap-frozen in liquid nitrogen for 30 min. Extraction was performed using methanol:acetonitrile:water (2:2:1, *v*/*v*/*v*) with homogenization (50 Hz, 4 min) and sonication (5 min). The mixture was centrifuged at 12,000 rpm for 15 min at 4 °C, and the supernatant was dried and reconstituted in acetonitrile:water (1:1, *v*/*v*) for analysis.

Sugars were detected via GC-MS (Agilent 7890B-5977B) on a DB-5MS column after oxidation and silylation. Energy metabolites were analyzed via UHPLC-MS/MS (SCIEX 6500 QTRAP+) on a Waters Atlantis Premier BEH Z-HILIC column [47]. FFAs were quantified on a DB-FastFAME column after methylation, as described by Chiu and Kuo [48]. Detailed mass spectrometric parameters and retention times are listed in Supplementary Table S2.

The targeted metabolites included 15 sugars (maltose, D-xylulose, D-sorbitol, D-ribose, D-mannose, levoglucosan, inositol, D-glucuronic acid, D-galacturonic acid, D-galactose, D-fructose, sucrose, trehalose, glucose and D-arabinitol), 61 energy metabolites (L-aspartate, cyclic AMP, UDP-glcNAc, dTMP, uracil, ADP, inosine, adenine, fumaric acid, malic acid, orgininosuccinic-acid, guanosine, succinic acid, D-erythrose 4-phosphate, xylulose-5-phosphate, D-ribose 5-phosphate-disodium, D-fructose-6-phosphate, D-ribulose-5-phosphate, sedoheptulose-7-phosphate, dihydroxyacetone-phosphate, glycerol-3-phosphate, phosphoenolpyruvic acid, 6-phosphogluconic-acid, 3-phenyllactic acid, phosphorylethanolamine, dAMP, glyceraldehyde-3-phosphate, tyrosine, lysine, serine, L-glutamic acid, threonine, DL-glyceric-acid, glucuronic-acid, L-leucine, gluconate, arginine, L-alanine, L-asparagine, L-2-hydroxyglutaric acid disodium, glutamine, D(+)-glucose, AMP, ATP, cysteic-acid, D-mannose-6-phosphate, pyruvic acid, L-cystine, flavin mononucleotide, 2-phospho-D-glyceric acid, nicotinamide-adenine-dinucleotide (NAD), lactate, citric-acid, 3-phosphoglycerate, dCMP, L-citrulline, c-di-AMP, acetyl-CoA, D-glucose-6-phosphate, ureidopropionate and cis-aconitic-acid), and 30 free fatty acids (FFAs) (eicosapentaenoic acid, gamma-linolenic acid, linoleic acid, linoelaidic acid, arachidonic acid, homo-gamma-linolenic acid, decanoic acid, docosanoic acid, nonadecanoic acid, octanoic acid, homo-gamma-linoleic acid, hexanoic acid, eicosanoic acid, octadecanoic acid, heptadecanoic acid, palmitic acid, pentadecanoic acid, tetradecanoic acid, tridecanoic acid, dodecanoic acid, undecanoic acid, tetracosanoic acid, tricosanoic acid, heneicosanoic acid, alpha-linolenic acid, oleic acid, palmitoleic acid, trans-oleic acid, gondoic acid, and erucic acid) (Detailed list provided in Supplementary Table S2).

### 2.8. Statistical Analysis

Experimental design and response surface modeling were conducted using Design-Expert v13.0 (Stat-Ease Inc., Minneapolis, MN, USA). Data were fitted to quadratic polynomial models and validated via ANOVA (*p* < 0.05). Biological assay data were arcsine square-root transformed [49] to normalize variance and analyzed via one-way ANOVA with Tukey’s HSD post hoc test using GraphPad Prism v8.0. Metabolomic data underwent multivariate analysis (PCA, OPLS-DA) [50,51] to identify differential biomarkers, with significance defined as VIP > 1.0 and *p* < 0.05. For univariate analysis of metabolite levels, as the data were non-normally distributed (Shapiro–Wilk test, *p* < 0.05), statistical comparisons were performed using the Kruskal–Wallis test followed by Dunn’s post hoc test. The optimization, yield validation, and biological quality assessment experiments were performed in two independent trials over time.

## 3. Results

### 3.1. Optimization of Fermentation Parameters via Response Surface Methodology

**Figure 1:**
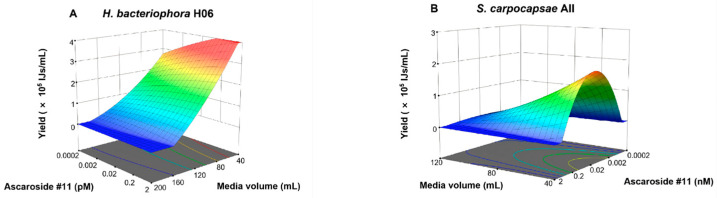
Three-dimensional response surface plots illustrating the interactive effects of ascaroside #11 concentration (Factor A) and media volume (Factor C) on infective juvenile (IJ) yield. (**A**): *Heterorhabditis bacteriophora* H06. (**B**): *Steinernema carpocapsae* All. The vertical axis represents IJ yield (×10^5^ IJs/mL), and horizontal axes display actual experimental values. Note: During the plotting of these 3D surfaces, the remaining experimental variables, DMSO concentration (Factor B) and inoculum size (Factor D), were mathematically held constant at their central design levels (0). For *S. carpocapsae* All (Panel B), the plotting range for media volume is restricted to 40–120 mL to highlight the valid optimization region.

Based on the central composite rotatable design in Table 1, the data analysis revealed distinct response patterns for the two nematode species, providing predictive models for yield maximization (Figure 1). The complete experimental design matrix, including actual and predicted yields for all 36 flasks, is provided in Supplementary Table S1.

**Figure 2:**
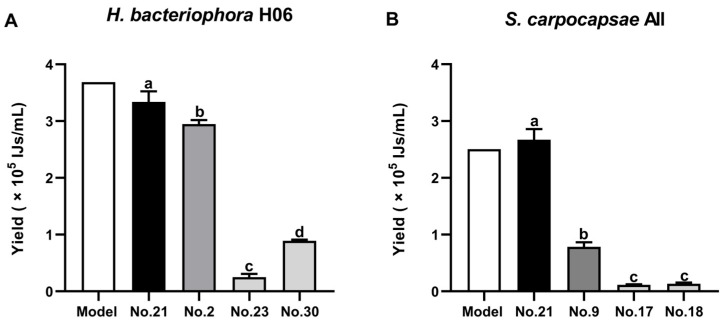
Validation of optimized liquid culture conditions: comparison of model-predicted values versus experimental yields. (**A**): *Heterorhabditis bacteriophora* H06. (**B**): *Steinernema carpocapsae* All. The “Model” bar represents the theoretical yield predicted by Response Surface Methodology (RSM). The bar labeled “No. 21” represents the experimental validation under these optimized conditions, while other numbered bars represent actual experimental yields from representative runs across the design space. Data for experimental runs are expressed as means ± SEM of three independent biological replicates (n = 3), whereas the Model value is presented as a single predicted point. Statistical analysis among experimental groups was performed using one-way ANOVA (*H. bacteriophora* H06: *F*_3,8_ = 557.5, *p* < 0.0001; *S. carpocapsae* All: *F*_3,8_ = 376.9, *p* < 0.0001) followed by Tukey’s HSD post hoc test. Different lowercase letters above bars indicate significant differences (*p* < 0.05).

The yield data for *H. bacteriophora* H06 fitted a quadratic model with high significance (*F* = 48.78, *p* < 0.0001) and a high coefficient of determination (*R*^2^ = 0.9572) (Supplementary Figure S1A,B). The lack-of-fit was non-significant (*p* = 0.1461), indicating the model accurately represented the biological variation within the experimental design space (Table 2). The dominant factor driving yield was media volume (C) (*F* = 491.32), confirming that oxygen availability is the primary constraint in liquid culture. Crucially, a significant interaction was observed between ascr#11 and media volume (AC). Response surface plots (Figure 1A) revealed that ascr#11 effectively enhanced yield only at low media volumes (40 mL). In high-volume (hypoxic) conditions, the pheromone effect was negligible. This suggests a hierarchical regulation where physiological oxygen status gates the response to developmental signaling. Based on the model optimization, the optimal conditions for *H. bacteriophora* H06 were determined to be Flask No. 21 (0.02 pM ascr#11, 0.01% DMSO, 40 mL media, 6000 IJs/mL inoculum). Experimental validation yielded 3.35 ± 0.29 × 10^5^ IJs/mL in Flask No. 21, closely matching the model prediction (3.69 × 10^5^ IJs/mL) (Figure 2A; Supplementary Table S1). Comparatively, this optimized yield was significantly higher than the second-highest-yield Flask No. 2 (0.002 pM ascr#11, 0.005% DMSO, 80 mL media, 12,000 IJs/mL inoculum) and the representative low-yield groups Flasks No. 23 (0.02 pM ascr#11, 0.01% DMSO, 120 mL media, 1500 IJs/mL inoculum) and No. 30 (0.02 pM ascr#11, 0.01% DMSO, 120 mL media, 6000 IJs/mL inoculum) (Figure 2A; Supplementary Table S3). Reproducibility of this optimized yield was confirmed in a second independent trial (Supplementary Figure S2A).

For *S. carpocapsae* All, the ANOVA results indicate that a reduced quadratic model was generated after eliminating non-significant main-effect terms (DMSO and inoculum size) (Table 3). The model was statistically significant (*R*^2^ = 0.9313, *p* < 0.0001). However, the ANOVA indicated a significant lack of fit (*p* < 0.0001) and a substantial discrepancy between the adjusted *R*^2^ (0.9171) and the predicted *R*^2^ (0.2382). Unlike the hermaphroditic *H. bacteriophora* H06, the amphimictic *S. carpocapsae* exhibited high experimental variability specifically at the high-volume boundary (coded value = +2 level, 200 mL). This boundary-specific stochasticity, likely associated with mating challenges under hypoxic conditions, created data noise that resulted in the calculated lack of fit. Consequently, the model may generate mathematical artifacts (overestimation) in this non-optimal region. Therefore, as opposed to asserting a universally robust optimum across the entire design space, we interpret this model as successfully identifying a promising optimization region that would benefit from further quantitative validation. Furthermore, graphical analysis was focused on the biologically reliable region (Figure 1B). Importantly, within this specific promising region, the model demonstrated high practical reliability: the optimal conditions were determined as Flask No. 21 (0.02 nM ascr#11, 0.01% DMSO, 40 mL media, 6000 IJs/mL inoculum). Under these conditions, the experimentally validated yield (2.67 ± 0.18 × 10^5^ IJs/mL) tightly matched the model’s predicted maximum of 2.50 × 10^5^ IJs/mL (Figure 2B; Supplementary Table S1). Residual analysis plots confirmed the normality of errors and the absence of systematic bias (Supplementary Figure S1C,D). Similarly to *H. bacteriophora* H06, media volume was the primary driver. The significant interaction term A^2^C reinforced the finding that pheromone efficacy is strictly dependent on oxygenation status. Similar consistent results were obtained in the independent validation trial (Supplementary Figure S2C). Comparatively, this optimized yield was significantly higher than the second-highest yield Flask No. 9 (0.2 nM ascr#11, 0.005% DMSO, 80 mL media, 3000 IJs/mL inoculum) and the representative low-yield groups Flasks No. 17 (0.0002 nM ascr#11, 0.01% DMSO, 120 mL media, 6000 IJs/mL inoculum) and No. 18 (2 nM ascr#11, 0.01% DMSO, 120 mL media, 6000 IJs/mL inoculum) (Figure 2B; Supplementary Table S3). Notably, the requirement for nM concentrations of ascr#11 in *Steinernema* (vs. pM in *Heterorhabditis*) highlights a significant interspecific difference in pheromone sensitivity.

### 3.2. Biological Quality Assessment

To verify if yield maximization compromised quality, we compared the “High Yield” optimized Flask No. 21 against solid culture control (CK) and representative non-optimal liquid variants, specifically the “Sub-optimal” (*H. bacteriophora* H06 No. 2; *S. carpocapsae* All No. 9) and “Low Yield” (*H. bacteriophora* H06 No. 23, No. 30; *S. carpocapsae* All No. 17, No. 18) groups.

**Figure 3:**
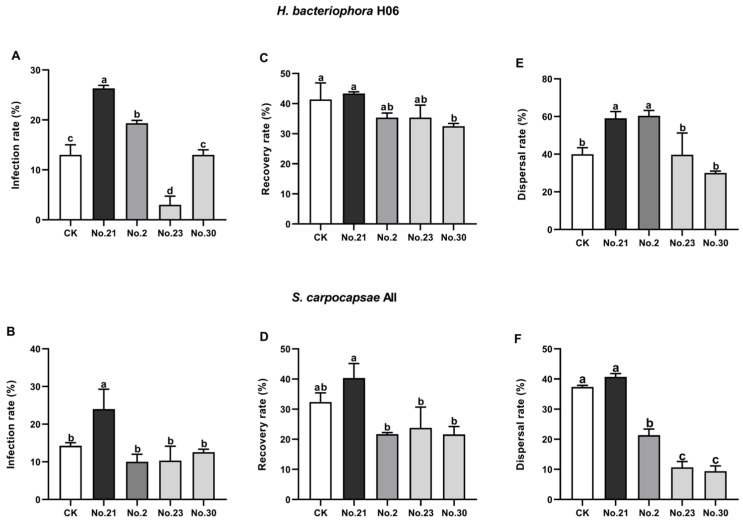
Biological quality and behavioral assessment of *Heterorhabditis bacteriophora* H06 and *Steinernema carpocapsae* All infective juveniles (IJs) produced in liquid culture. (**A**,**B**): infectivity against *Galleria mellonella* larvae (96 h post-infection). (**C**,**D**): Recovery rate of IJs induced in bacteria-conditioned media. (**E**,**F**): Dispersal rate on 2% agar plates. Treatments: “No. 21” represents the optimized liquid culture condition; “No. 2”, “No. 9”, “No. 17”, “No. 18”, “No. 23”, “No. 30” represent comparative liquid culture runs across the design space; “CK” represents the solid culture control. Statistics: Data are expressed as means ± SEM (n = 3 biological replicates). Statistical differences were determined by one-way ANOVA ((**A**): *F*_4,10_ = 79.89, *p* < 0.0001; (**B**): *F*_4,10_ = 10.23, *p* = 0.0015; (**C**): *F*_4,10_ = 6.112, *p* = 0.0094; (**D**): *F*_4,10_ = 11.63, *p* = 0.0009; (**E**): *F*_4,10_ = 15.68, *p* = 0.0003; (**F**): *F*_4,10_ = 252.0, *p* < 0.0001) followed by Tukey’s HSD post hoc test. Different lowercase letters above bars indicate statistical significance (*p* < 0.05).

Surprisingly, optimized liquid cultures exhibited superior infectivity. The single-IJ infection rate of *H. bacteriophora* H06 from Flask No. 21 reached 26%, and the sub-optimal group (No. 2) reached 19%, both significantly higher than the solid control (13%) (*p* < 0.05). In sharp contrast, the low-yield group (No. 23) suffered a drastic loss of pathogenicity, with the infection rate dropping to only 3.3% (Figure 3A). Similarly, the IJs of *S. carpocapsae* All from Flask No. 21 achieved 24% *Galleria* larval infectivity, significantly outperforming both the solid control (14%) and the non-optimal groups (No. 9, 17, 18), which ranged between 10% and 12.5% (Figure 3B). These trends of superior infectivity were consistently observed for both species in the second trial (Supplementary Figure S2B,D).

Recovery rates of the IJs of *H. bacteriophora* H06 from Flask No. 21 (43.3%), solid culture (41.3%) and other flask numbers were not statistically different, except from Flask No. 30 (the lower yield culture flask), which dropped to 32.5% (Figure 3C). IJ recovery rates of *S. carpocapsae* All from Flask No. 21 (approximately 40%) and solid culture (32.3%) did not exhibit significant differences, but IJ recovery rates of *S. carpocapsae* All from Flask No. 21 showed differed significantly from the other liquid culture groups (Figure 3D).

The dispersal indices showed that the *H. bacteriophora* H06 IJs from the culture flask containing the higher yield exhibited the strongest dispersal ability compared to those from the culture flasks containing lower IJ yields (Figure 3E). The similar patterns were found in *S. carpocapsae* All IJs, except that no significant difference was detected between Flask No. 21 (41%) and solid culture (37.3%) (Figure 3F). These results may reflect variable physiological health and energy reserves in the IJs from different cultures. Detailed statistical analysis tables (ANOVA) for all biological assays are listed in Supplementary Table S3.

### 3.3. Metabolic Characterization Associated with the High-Quality Phenotype

To characterize the metabolic profiles behind the simultaneous increase in yield and quality, we performed targeted metabolomics of sugars, energy metabolites and free fatty acids on *H. bacteriophora* H06 IJs. We compared the “High Yield” group (Flask No. 21), the “Low Yield” group (Flask No. 23), and the solid culture control (CK).

**Figure 4:**
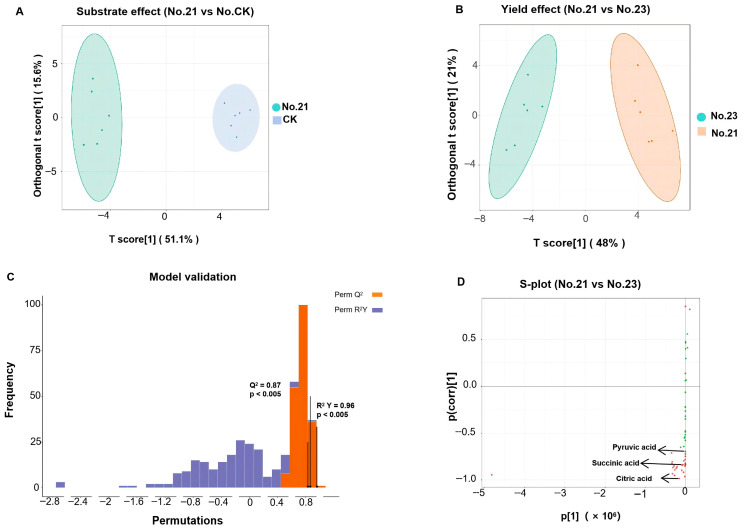
Multivariate statistical analysis of the hydrophilic metabolic profiles (focusing on central carbon and energy metabolism) of *Heterorhabditis bacteriophora* H06 derived from UHPLC-MS/MS analysis. (**A**): OPLS-DA score plot comparing the optimized liquid culture (No. 21, High Yield; green ellipses) versus the solid culture control (CK; blue ellipses). (**B**): OPLS-DA score plot differentiating the High Yield group (No. 21; green ellipses) from the Low Yield group (No. 23; orange ellipses). In both panels (**A**,**B**), the shaded ellipses represent the 95% confidence interval (CI). (**C**): Permutation test results (n = 200) validating the OPLS-DA model in Panel (**B**), showing high predictivity (*Q*^2^ = 0.87) and goodness of fit (*R*^2^*Y* = 0.96) with statistical significance (*p* < 0.005). (**D**): S-plot generated from the model in Panel (**B**), identifying key polar biomarkers contributing to the separation between high- and low-yield groups. Red dots represent differential metabolites identified based on VIP > 1.0 and *p* < 0.05. The green dots represent non-significant metabolites that did not meet the selection criteria (VIP ≤ 1 or *p* ≥ 0.05).

Hydrophilic metabolites were analyzed via UHPLC-MS/MS to assess the global state of central carbon and energy metabolism. The OPLS-DA score plots showed clear separation between the groups. There was a clear distinction between the high-yield liquid group (Flask No. 21) and the solid control (CK) (Figure 4A). Crucially, within the liquid cultures, the high-yield group (Flask No. 21) was significantly separated from the low-yield group (Flask No. 23) (Figure 4B). The statistical robustness of this model was validated by permutation tests (n = 200), which showed high predictivity (*Q*^2^ = 0.87) and no overfitting (Figure 4C). Furthermore, the specific metabolic features driving this separation were identified using the S-plot (Figure 4D), revealing potential biomarkers associated with yield optimization. This indicates that yield differences are accompanied by systemic changes in the metabolic network. A similar segregation trend was observed in the hydrophobic lipidomic profile, as shown in Supplementary Figure S3.

**Figure 5:**
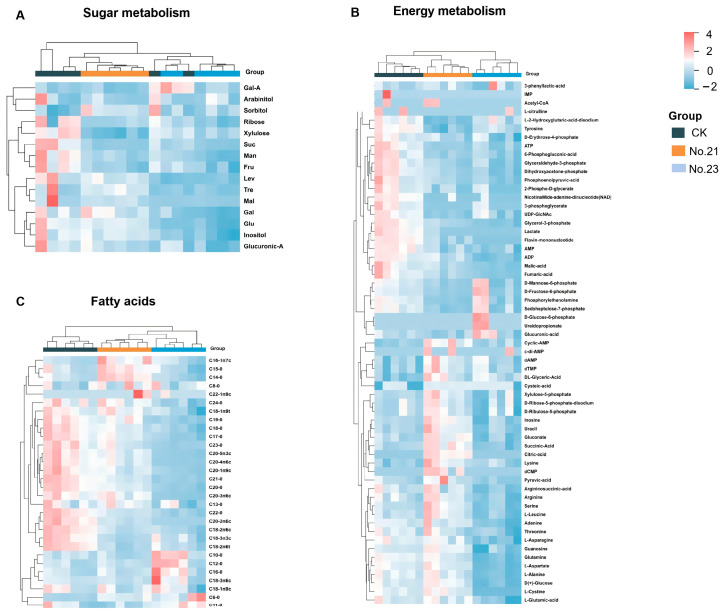
Targeted metabolomic profiling reveals metabolic alignment in *Heterorhabditis bacteriophora* H06 infective juveniles produced under optimized liquid culture conditions. Heatmaps visualize the relative abundance of differential metabolites across three distinct culture groups. Data were Z-score normalized by row, and samples (columns) were clustered using Euclidean distance to demonstrate metabolic similarity. (**A**): Sugar and polyol metabolism, illustrating the accumulation of the stress-protectant trehalose. (**B**): Energy metabolism intermediates (e.g., Citric acid, Pyruvic acid), showing active central carbon metabolism. (**C**): Free fatty acid profiles, highlighting the accumulation of essential polyunsaturated fatty acids (PUFAs) such as EPA and ARA. Groups: CK represents the solid culture control (grey bar, n = 6); No. 21 represents the optimized high-yield liquid culture (40 mL media + ascr#11, orange bar, n = 6); No. 23 represents the low-yield liquid culture (120 mL media, blue bar, n = 6). The color scale ranges from blue (downregulated) to red (upregulated).

To systematically visualize metabolic differences across the IJ groups, we performed hierarchical clustering analysis on three distinct metabolic modules: sugar metabolism (Figure 5A), energy metabolism (Figure 5B), and fatty acids (Figure 5C). The heatmaps consistently demonstrate that the metabolic fingerprint of the high-yield group (IJs from Flask No. 21) is distinct from the low-yield group (IJs from Flask No. 23) and clusters closely with the solid control (CK) across all three categories. This clear separation suggests a metabolic shift where the synergistic application of reduced media volume (high oxygen availability) and ascr#11 resembled the metabolic phenotypes of high-yield group (No. 21) as the metabolic baseline from solid culture, a state characterized by high energy flux and stress tolerance that was not achievable under standard low-yield liquid culture conditions.

**Figure 6:**
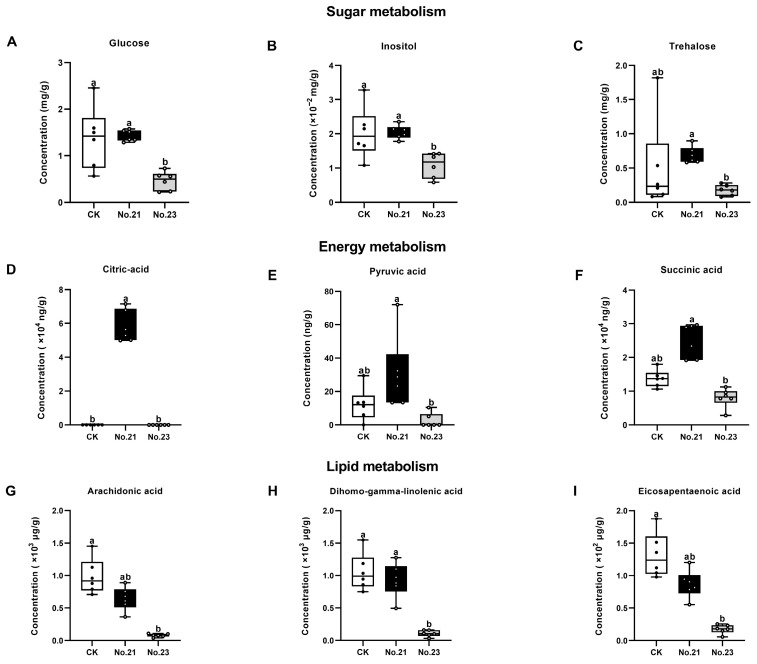
Quantitative analysis of key differential metabolites in *Heterorhabditis bacteriophora* H06 infective juveniles across different culture conditions. Panels display the concentration levels of (**A**–**C**) sugar metabolism-related compounds: (**A**): Glucose, (**B**): Inositol, and (**C**): Trehalose. (**D**–**F**) TCA cycle and energy metabolism intermediates: (**D**): Citric acid, (**E**): Pyruvic acid, and (**F**): Succinic acid, and (**G**–**I**) polyunsaturated fatty acids: (**G**): Arachidonic acid, (**H**): Dihomo-gamma-linolenic acid, and (**I**): Eicosapentaenoic acid. (**A**–**I**) Box-and-whisker plots overlaid with jitter points representing individual biological replicates (n = 6). The horizontal line within the box indicates the median, the box boundaries represent the 25th and 75th percentiles (interquartile range), and the whiskers extend to the minimum and maximum values. Treatments: “CK” represents the solid culture control; “No. 21” represents the optimized high-yield liquid culture condition; “No. 23” represents the low-yield liquid culture condition. Statistics: Statistical significance was determined using the Kruskal–Wallis test followed by Dunn’s multiple comparisons test for all metabolites. Different lowercase letters above the boxes indicate statistically significant differences (*p* < 0.05).

Sugar metabolism. Comparative analysis of high-yield (No. 21) and low-yield (No. 23) groups, under strict screening criteria (VIP > 1, *p* < 0.05, FC > 2), identified 3 significantly enriched metabolites among the 15 quantified sugars. These key upregulated metabolites were identified as trehalose (FC = 3.93), maltose (FC = 3.75), and glucose (FC = 3.11). While maltose exhibited substantial accumulation, reflecting active glycogen mobilization, for the graphical visualization we prioritized inositol (Figure 6B) alongside glucose (Figure 6A) and trehalose (Figure 6C). This selection was driven by the specific roles of inositol and trehalose as critical stress-signaling biomarkers, which are more indicative of IJ fitness than metabolic intermediates like maltose. Notably, trehalose levels in the low-yield group were significantly lower than those in the solid culture control (CK). However, in the high-yield group, trehalose content was restored to levels statistically comparable to the CK group (*p* > 0.05) (Figure 6C). No significant differences were observed in the levels of the remaining sugars, such as D-xylulose and D-ribose, between the IJs from No. 21 and No. 23, as detailed in the comprehensive profile (Supplementary Figure S4).

Energy metabolism. Targeted analysis of 61 energy-related metabolites (Supplementary Table S2) revealed a broad upregulation of central metabolic pathways in the high-yield group (No. 21) compared to the low-yield group (No. 23). Under strict screening criteria (VIP > 1, *p* < 0.05, FC > 2), 12 metabolites were identified as significantly enriched. These included pyruvic acid, succinic acid, citric acid, L-cystine, argininosuccinic acid, dCMP, gluconate, guanosine, L-aspartate, glutamine, uracil, and L-alanine. Among these, pyruvic acid exhibited the most dramatic elevation (FC = 11.87), followed by argininosuccinic acid (FC = 3.41). However, to characterize the central carbon flux and mitochondrial respiratory status, we focused our quantitative analysis on citric acid (Figure 6D), pyruvic acid (Figure 6E), and succinic acid (Figure 6F) as they represent the rate-limiting nodes of glycolysis and the TCA cycle. Furthermore, citric acid was exclusively detected in the high-yield group (No. 21). Succinic acid (FC = 3.11) was also markedly higher compared to No. 23. It is worth noting that L-cystine and dCMP were also exclusively detected in the high-yield group. The complete profile of energy metabolites is visualized in Supplementary Figure S5.

Lipid metabolism. Targeted lipidomic profiling quantified 30 free fatty acids (FFAs) (Supplementary Table S2). Comparative analysis of the high-yield (No. 21) and low-yield (No. 23) groups, under strict screening criteria (VIP > 1, *p* < 0.05, FC > 2), identified 8 significantly enriched fatty acids. The enriched profile included polyunsaturated fatty acids (PUFAs) such as dihomo-gamma-linolenic acid (DGLA), arachidonic acid (ARA), eicosapentaenoic acid (EPA), and cis-11,14-eicosadienoic acid, as well as saturated and monounsaturated fatty acids including eicosanoic acid, tetradecanoic acid, heneicosanoic acid, and cis-11-eicosenoic acid. Among these, we specifically prioritized the top-three PUFAs—ARA (Figure 6G), DGLA (Figure 6H), and EPA (Figure 6I)—for detailed visualization. This selection was based on their status as primary bioactive lipids, distinct from general structural fatty acids. The levels of these functional lipids in the low-yield group (No. 23) were significantly lower than those in the solid culture control (CK). Conversely, in the high-yield group (No. 21), their concentrations were elevated to levels not significantly different from the CK group (*p* > 0.05). The global lipidomic changes are presented in Supplementary Figure S6.

**Figure 7:**
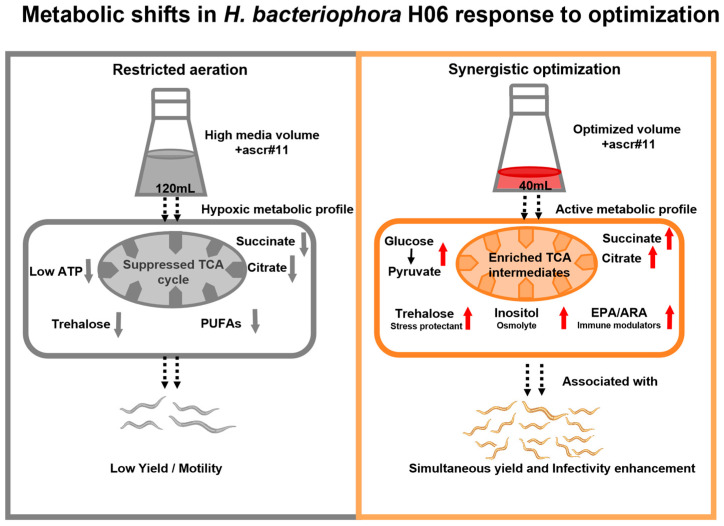
Schematic representation of the metabolic shifts in *Heterorhabditis bacteriophora* H06 associated with synergistic optimization. The diagram summarizes the observed metabolic differences between the restricted aeration condition (Left, Low Yield) and the optimized synergistic strategy (Right, High Yield). (**Left**): The central design point condition (120 mL media volume combined with non-optimized ascr#11 levels) results in restricted aeration, coinciding with a metabolic profile characterized by suppressed mitochondrial TCA cycle activity and downregulated biosynthesis of stress protectants. (**Right**): Synergistic optimization (40 mL media volume combined with optimally matched ascr#11) creates a permissive environment with improved aeration, which is associated with upregulated central carbon metabolism (glucose, pyruvate), enhanced TCA cycle activity (succinate, citrate), and the accumulation of fitness-related biomarkers including trehalose, inositol, and PUFAs (EPA/ARA). Note: This figure represents a conceptual model based on metabolomic (n = 6) and phenotypic profiles. Media volume serves as an established proxy for aeration status in standard shake-flask fermentations. Arrows indicate inferred metabolic associations, with causal validation reserved for future investigation. Gray represents the low‑yield condition (restricted aeration); orange represents the optimized high‑yield condition (improved aeration). Dashed arrows indicate inferred metabolic associations, with causal validation reserved for future investigation.

The integration of metabolomic data and fermentation parameters is summarized in Figure 7. The data suggest that under high-volume conditions, oxygen limitation coincides with suppressed mitochondrial metabolism. Conversely, the synergistic optimization of reduced media volume and ascr#11 application is associated with elevated levels of energy metabolites, carbohydrate reserves (trehalose), and specific PUFAs, correlating with the observed high-yield and high-fitness phenotype.

## 4. Discussion

While liquid fermentation enables commercial-scale production, optimizing biological quality in high-density cultures remains a critical priority [3,8]. High-yield processes are frequently associated with depleted lipid reserves and diminished infectivity, a phenomenon largely attributed to metabolic stress under oxygen-limited conditions [10,35]. Consequently, yields for most commercial species (typically *Heterorhabditis* spp. and *Steinernema* spp.) in liquid culture systems remain constrained to 1–3 × 10^5^ IJs/mL [52,53], underscoring the persistent difficulty of simultaneously improving production intensity and biological fitness.

This study demonstrates that these limitations can be effectively addressed through precise process engineering. Rather than being an inherent biological trait, the compromised quality often observed in mass production appears to be a consequence of environmental limitations. By identifying the synergistic interaction between physical parameters (media volume/OTR) and chemical signaling (ascr#11), we established a protocol that promotes the concurrent improvement of both traits. While dimethyl sulfoxide (DMSO) was initially included in the optimization matrix as a potential chemical chaperone based on previous findings [26,30], our statistical models indicate that its individual contribution is relatively minor compared to the dominant effects of aeration and ascr#11. Within the optimized system, DMSO is therefore better interpreted as a secondary or context-dependent factor, which may provide subtle supportive effects (e.g., influencing solubility or membrane permeability), rather than serving as a primary driver of the enhanced phenotype. The reduction in media volume, functioning as a proxy for improved aeration, is inferred to relieve the primary metabolic constraint of hypoxia [7,54], creating a permissive environment where pheromone signaling (ascr#11) can effectively coordinate developmental synchronization. Under these optimized conditions, the yield of *H. bacteriophora* reached 3.35 × 10^5^ IJs/mL. This is comparable to the high yields reported in complex fed-batch systems (3.6–4.2 × 10^5^ IJs/mL) [55] and represents the cutting edge of current fermentation technology [52]. Crucially, this system induced a “high-yield/high-fitness phenotype” with infectivity significantly exceeding that of the solid culture controls used in this study. These results suggest that synergistically optimized liquid cultures are not merely capable of matching traditional methods but have the potential to surpass them in specific fitness traits, thereby establishing a new benchmark for biological quality in in vitro mass production [56]. It should be noted that in the present study, our assessment of “biological quality” primarily refers to the physiological and functional status of IJs at the point of harvest. While these metrics indicate an enhanced potential for long-term storage, they do not substitute for empirically validated shelf-life.

In this study, the optimized shake-flask protocol successfully established the phenotypic proof-of-concept for the synergy of ascr#11 and improved aeration on the yield and fitness of entomopathogenic nematodes. Although the standardized shake-flask system is widely accepted as a standard practice for initial multifactorial screening [57,58], relying on media volume as a proxy for aeration limits the ability to obtain continuous, quantitative dissolved oxygen (DO) data. For industrial scale-up, transitioning from initial shake-flask screening to instrumented bioreactor systems is essential in quantifying exact oxygenation kinetics [10,52]. Therefore, future studies utilizing advanced microbioreactor systems (such as the ambr^®^ system) with real-time DO monitoring are highly warranted to definitively quantify the dynamic synergy between real-time oxygenation kinetics and ascr#11 signaling.

The metabolomic analysis reveals that the “high-yield/high-fitness phenotype” of *H. bacteriophora* is characterized by a distinct biochemical profile that closely aligns with the solid-culture baseline (Figure 5). This suggests that the optimized liquid process effectively alleviates the metabolic suppression typically imposed by hypoxic conditions. While metabolomic profiling was exclusively conducted on *H. bacteriophora*, providing robust mechanistic support for this species, we cautiously interpret these biochemical shifts as a plausible explanatory framework for the parallel phenotypic enhancements observed in *S. carpocapsae*. Given the evolutionary conservation of core metabolic pathways between *H. bacteriophora* and the model organism *C*. *elegans* [59], established metabolic traits in *C. elegans* provide a valuable interpretive framework for these observations.

The accumulation of specific carbohydrates indicates a physiological state pre-adapted for storage and stress tolerance. The significant 3.93-fold elevation of trehalose aligns with the “water replacement hypothesis,” a well-documented mechanism for desiccation tolerance in both *C. elegans* [60] and EPNs [15]. This enrichment provides a plausible biochemical basis for the stability of the IJs produced in our optimized system. Although empirical long-term storage assays (e.g., ≥2 months) are ultimately required to definitively establish commercial shelf-life, the restoration of this critical stress protectant to levels comparable to solid-culture controls provides a robust physiological indicator for enhanced storage potential. Furthermore, the high microbial biomass generated during liquid culture can lead to rapid oxygen depletion if left untreated. In our methodology, thorough post-harvest washing was employed to remove bacterial interference prior to our immediate biological assessments. Importantly, if these liquid-cultured IJs are intended for long-term storage, this rigorous washing step is conceptually critical to arrest microbial oxygen consumption and preserve IJ longevity. Concurrently, the elevated levels of inositol—a precursor for IP3 signaling—are consistent with the requirements for neuromuscular function. In *C. elegans*, inositol signaling is integral to regulating rhythmic contractile activities [61], a physiological prerequisite that likely supports the superior motility and dispersal capacity observed in our optimized IJs (Figure 3E).

The synchronous elevation of organic acids and amino acids reflects a robust aerobic metabolism and redox homeostasis. The specific enrichment of TCA cycle intermediates (most notably the dramatic 11.87-fold elevation of pyruvate) implies a shift from anaerobic glycolysis to mitochondrial respiration. For instance, in *C. elegans*, succinate transport is a rate-limiting step for Complex II-mediated respiration [17], suggesting that the succinate accumulation in the optimized IJs is indicative of unblocked respiratory flux. Additionally, the exclusive detection of L-cystine, a precursor for glutathione [62], suggests that the optimized fermentation environment maintains essential biosynthetic capacities.

The lipid profile provides a biochemical rationale for the enhanced pathogenicity. The high levels of long-chain PUFAs (EPA and ARA) observed in the optimized group coincide with the reactivation of oxygen-dependent Δ 5-desaturases [16], which are typically substrate-limited in hypoxic cultures. This specific lipid composition is biologically significant: EPA has been associated with the regulation of infectivity-related behaviors in EPNs [63], while ARA serves as a precursor for eicosanoids involved in insect immune modulation [36]. The presence of these functional lipids is consistent with the superior infectivity rates observed in the bioassays (Figure 3A). Furthermore, while the targeted metabolomic profiling reveals a distinct biochemical signature associated with the optimized liquid culture conditions, we acknowledge that these findings establish a correlation rather than definitive causality. The observed metabolic changes are consistent with known physiological roles of key metabolites (e.g., trehalose in stress tolerance and PUFAs in host–pathogen interactions), providing a plausible biochemical context for the enhanced IJ fitness. However, future functional validations, such as targeted gene knockouts or specific metabolic pathway interventions, are required to conclusively determine the direct causal roles of these metabolites.

## 5. Conclusions

In conclusion, this study demonstrates that the persistent limitation regarding the simultaneous enhancement of yield and quality in liquid fermentation can be effectively addressed through the synergistic optimization of media volume (inferred to represent oxygen availability) and ascaroside signaling. Specifically for *H. bacteriophora*, our targeted metabolomic analysis reveals that this synergy does not merely increase biomass but substantially improves the IJ physiological state. This improvement is supported by a metabolic profile comparable to the robust baseline of solid-cultured nematodes, specifically through the reactivation of the mitochondrial TCA cycle and the accumulation of fitness-related metabolites, including trehalose and functional polyunsaturated fatty acids (EPA and ARA). Together, these metabolic findings offer a plausible explanatory framework for the generalized yield and quality enhancements observed across both nematode species. These findings establish a scalable paradigm for the industrial mass production of high-quality biocontrol agents, confirming that high fermentation efficiency is compatible with superior biological efficacy.

## Figures and Tables

**Table 1:** Experimental factors and levels used in the central composite rotatable design (CCRD).

Factors	Symbol	Coded Levels				
	Factor A	−2	−1	0	1	2
Ascr#11 for *H. bacteriophora* H06 (pM)		0.0002	0.002	0.02	0.2	2
Ascr#11 for *S. carpocapsae* All (nM)		0.0002	0.002	0.02	0.2	2
DMSO (% *v*/*v*)	Factor B	0.0025	0.005	0.01	0.02	0.04
Media volume (mL/flask)	Factor C	40	80	120	160	200
Inoculum size (IJs/mL)	Factor D	1500	3000	6000	12,000	24,000

Note: Factors A–D represent the independent variables used in the design. “H06” and “All” denote the specific nematode strains evaluated. Abbreviations: ascr#11: Ascaroside #11; DMSO: dimethyl sulfoxide; IJs: infective juveniles.

**Table 2:** Analysis of Variance (ANOVA) for the Quadratic Model of *Heterorhabditis bacteriophora* H06 Yield.

Source	*df*	*F*-Value	*p*-Value
Model	11	48.78	<0.0001
A-ascr	1	6.38	0.0185
B-DMSO	1	0.2022	0.6570
C-Media volume	1	491.32	<0.0001
D-Inoculum size	1	9.02	0.0062
AC	1	6.83	0.0152
C^2^	1	10.40	0.0036
D^2^	1	8.22	0.0085
Residual	24		
Lack of Fit	13	1.90	0.1461

Note: *R*^2^ = 0.9572; adjusted *R*^2^ = 0.9376; predicted *R*^2^ = 0.8598; Adeq Precision = 31.35; C.V. = 19.84%. Significant terms (*p* < 0.05) are highlighted in bold. ns: not significant.

**Table 3:** Analysis of Variance (ANOVA) for the Reduced Quadratic Model of *Steinernema carpocapsae* All Yield.

Source	*df*	*F*-Value	*p*-Value
Model	6	65.56	<0.0001
A-ascr	1	0.0209	0.8870
C-Media volume	1	207.23	<0.0001
AC	1	0.0005	0.9826
A^2^	1	2.38	0.1339
C^2^	1	133.13	<0.0001
A^2^C	1	58.48	<0.0001
Residual	29		
Lack of Fit	18	23.52	<0.0001
Pure Error	11		
Cor Total	35		

Note: *R*^2^ = 0.9313; adjusted *R*^2^ = 0.9171; predicted *R*^2^ = 0.2382; Adeq Precision = 46.17; C.V. = 52.01%. Factors B (DMSO concentration) and D (Inoculum size) were non-significant and removed from the model. The term A^2^C was significant, requiring the retention of lower-order parent terms (A, AC, A^2^) to maintain model hierarchy.

## Data Availability

All data generated or analyzed during this study are included in this published article and its Supplementary Materials.

## Supplementary Materials

The following supporting information can be downloaded at: https://www.mdpi.com/article/10.3390/life16050703/s1, Supplementary Table S1: Experimental design matrix (CCRD) and response values for *H. bacteriophora* H06 and *S. carpocapsae* All; Supplementary Table S2: Targeted metabolomic parameters and metabolite list (Excel file); Supplementary Table S3: Detailed one-way ANOVA results for experimental validation and biological quality assessments; Supplementary Table S4: Statistical analysis of differential metabolites in *H. bacteriophora* H06; Supplementary Figure S1: Diagnostic plots for the Response Surface Methodology (RSM) models; Supplementary Figure S2: Validation of yield and infectivity in a second independent experimental trial; Supplementary Figure S3: Multivariate statistical analysis of the hydrophobic lipidomic profiles; Supplementary Figure S4: Comprehensive metabolic profile of sugars and polyols in *H. bacteriophora* H06; Supplementary Figure S5: Comparative analysis of TCA cycle intermediates and energy-related metabolites; Supplementary Figure S6: Global profiling of free fatty acids (FFAs) and lipid composition.

## Abbreviations

The following abbreviations are used in this manuscript:

ANOVA	Analysis of variance
ARA	Arachidonic acid
ascr#11	Ascaroside #11
CCRD	Central composite rotatable design
DGLA	Dihomo-gamma-linolenic acid
DMSO	Dimethyl sulfoxide
DO	Dissolved oxygen
EPA	Eicosapentaenoic acid
EPNs	Entomopathogenic nematodes
FC	Fold change
FFAs	Free fatty acids
GC-MS	Gas chromatography–mass spectrometry
IJs	Infective juveniles
NBTA	Nutrient agar supplemented with triphenyl tetrazolium chloride and bromothymol blue
OPLS-DA	Orthogonal partial least squares discriminant analysis
OTR	Oxygen transfer rate
PCA	Principal component analysis
PUFAs	Polyunsaturated fatty acids
SEM	Standard error of the mean
TCA	Tricarboxylic acid
UCP4	Uncoupling protein 4
UHPLC-MS/MS	Ultra-high-performance liquid chromatography–tandem mass spectrometry
VIP	Variable importance in projection
