# Supplementary material for: Synergy of Ascr#11 and Improved Aeration Drives Enhanced Yield and Fitness of Entomopathogenic Nematodes
Source: Life (Basel). 2026 Apr 22;16(5):703. doi: 10.3390/life16050703 (PMC13208442; doi:10.3390/life16050703)
Supplement: Supplementary file 1 [file life-16-00703-s001.zip › life-4266674-supplementary.pdf]

Supplementary Materials

Synergy of Ascr#11 and Improved Aeration Drives Enhanced Yield and Fitness of Entomopathogenic Nematodes

Qiji Wang [1,2], Huilin Liao [1,2], Dzmitry Voitka [3], Alena Yankouskaya [3], Richou Han [2], Yongling Jin [1,*] and Li Cao [2,*]

[1] College of Agriculture, Heilongjiang Bayi Agricultural University, Daqing 163319, China
[2] Guangdong Key Laboratory of Animal Conservation and Resource Utilization, Guangdong Public Laboratory of Wild Animal Conservation and Utilization, Institute of Zoology, Guangdong Academy of Sciences, Guangzhou 510260, China
[3] Institute of Plant Protection, National Academy of Sciences of Belarus, Mira Str. 2, Ag. Priluki, Minsk District, 223011 Minsk, Minsk Region, Belarus
* Correspondence: gygjyl08@163.com (Y.J.); caol@giz.gd.cn (L.C.)

This file includes: Supplementary Table S1, Supplementary Table S3, Supplementary Table S4, Supplementary Figure S1, Supplementary Figure S2, Supplementary Figure S3, Supplementary Figure S4, Supplementary Figure S5, Supplementary Figure S6

Other Supplementary Materials for this manuscript include the following: Supplementary Table S2 (Excel file)

Supplementary Table S1. Experimental design matrix (Central Composite Rotatable Design) and response values for *Heterorhabditis bacteriophora* H06 and *Steinernema carpocapsae* All.

Run	Ascr#11 (X_1)	DMSO (X_2)	Medium volume (X_3)	Inoculum size (X_4)	Yield (*H. bacteriophora* H06) (10^5 IJs/mL)	Yield (*S. carpocapsae* All) (10^5 IJs/mL)
1	−1	−1	−1	−1	2.13	0.43
2	−1	−1	−1	+1	2.95	0.59
3	−1	−1	+1	−1	<0.01	0.03
4	−1	−1	+1	+1	<0.01	<0.01
5	−1	+1	−1	−1	2.30	0.33
6	−1	+1	−1	+1	2.67	0.55
7	−1	+1	+1	−1	<0.01	<0.01
8	−1	+1	+1	+1	0.01	<0.01
9	+1	−1	−1	−1	2.12	0.79
10	+1	−1	−1	+1	1.82	0.53
11	+1	−1	+1	−1	0.01	0.01
12	+1	−1	+1	+1	0.01	<0.01
13	+1	+1	−1	−1	1.60	0.36
14	+1	+1	−1	+1	2.19	0.25
15	+1	+1	+1	−1	0.01	0.02
16	+1	+1	+1	+1	0.02	0.02
17	−2	0	0	0	1.16	0.11
18	+2	0	0	0	0.92	0.14

Run	Ascr#11 (X_1)	DMSO (X_2)	Medium volume (X_3)	Inoculum size (X_4)	Yield (*H. bacteriophora* H06) (10^5 IJs/mL)	Yield (*S. carpocapsae* All) (10^5 IJs/mL)
19	0	−2	0	0	1.19	0.15
20	0	+2	0	0	1.08	0.18
**21**	**0**	**0**	**−2**	**0**	**3.35**	**2.67**
22	0	0	+2	0	<0.01	<0.01
23	0	0	0	−2	0.25	0.14
24	0	0	0	+2	1.16	0.26
25	0	0	0	0	1.00	0.13
26	0	0	0	0	1.08	0.12
27	0	0	0	0	1.18	0.14
28	0	0	0	0	1.13	0.09
29	0	0	0	0	1.04	0.15
30	0	0	0	0	0.89	0.09
31	0	0	0	0	0.89	0.10
32	0	0	0	0	1.24	0.11
33	0	0	0	0	1.31	0.11
34	0	0	0	0	0.95	0.10
35	0	0	0	0	1.02	0.16
36	0	0	0	0	0.93	0.17

Note: Experimental design matrix based on Central Composite Rotatable Design (CCRD) and observed yields for *Heterorhabditis bacteriophora* H06 and *Steinernema carpocapsae* All. The table displays the coded levels of the four independent variables: ascr#11 concentration (X_1), DMSO concentration (X_2), medium

volume (X_3), and inoculum size (X_4). The "Actual Yield" represents the mean value derived from three independent biological replicates (n = 3), each averaged from three technical counts. The center point (0, 0, 0, 0) was replicated twelve times to estimate pure experimental error and assess the reproducibility of the response surface models.While coded levels are identical, the actual concentrations for X_1 differ between species (pM for *H. bacteriophora* H06; nM for *S. carpocapsae* All), as detailed in Table 1 of the main text.

Supplementary Table S3. Detailed one-way ANOVA results for the experimental validation (Fig. 2) and biological quality assessments (Fig. 3).

Dataset / Parameter	Source of Variation	*df*	SS	MS	*F*-value	*P*-value
Fig. 2A: Yield (*H. bacteriophora* H06)	Treatment (Between)	3	20.08	6.893	646.8	< 0.0001
	Residual (Within)	8	0.0853	0.0107		
	Total	11	20.76			
Fig. 2B: Yield (*S. carpocapsae* All)	Treatment (Between)	3	13.07	4.355	427.5	< 0.0001
	Residual (Within)	8	0.0815	0.0102		
	Total	11	13.15			
Fig. 3A: Infection rate (*H. bacteriophora* H06)	Treatment (Between)	4	0.2261	0.0565	79.89	< 0.0001
	Residual (Within)	10	0.0071	0.0007		
	Total	14	0.2331			
Fig. 3B: Infection rate (*S. carpocapsae* All)	Treatment (Between)	4	395	98.75	10.23	0.0015
	Residual (Within)	10	96.6	9.66		
	Total	14	491.6			
Fig. 3C: Recovery rate (*H. bacteriophora* H06)	Treatment (Between)	4	250.3	62.57	6.112	0.0094
	Residual (Within)	10	102.4	10.24		
	Total	14	352.7			
Fig. 3D: Recovery rate (*S. carpocapsae* All)	Treatment (Between)	4	812.1	203	11.63	0.0009

	Residual (Within)	10	174.6	17.46		
	Total	14	986.7			
Fig. 3E: Dispersal rate (*H. bacteriophora* H06)	Treatment (Between)	4	2121	530.3	15.68	0.0003
	Residual (Within)	10	338.2	33.82		
	Total	14	2460			
Fig. 3F: Dispersal rate (*S. carpocapsae* All)	Treatment (Between)	4	2561	640.1	252	< 0.0001
	Residual (Within)	10	25.4	2.54		
	Total	14	2586			

Note: *df*: Degrees of Freedom; SS: Sum of Squares; MS: Mean Square. Data were analyzed after arc-sine square root transformation to meet the assumptions of normality and homogeneity of variance required for one-way ANOVA. For Fig. 2, statistical comparisons were performed among experimental groups (n = 3) only. The theoretical model prediction (n = 1) serves as a reference and was excluded from statistical testing.

Supplementary Table S4. Statistical analysis of differential metabolites in *H. bacteriophora* H06 (Fig. 6).

Metabolite (Panel)	Statistical Test	Statistic	*df*	*P*-value	Assumption Checka
		Sugar metabolism			
Glucose (Fig. 6A)	Kruskal–Wallis	H=10.19	2	0.0020	Normality: p<0.05
Inositol (Fig. 6B)	Kruskal–Wallis	H=9.696	2	0.0030	Normality: p<0.05
Trehalose (Fig. 6C)	Kruskal–Wallis	H=8.842	2	0.0063	Normality: p<0.05
		Energy metabolism			
Citric acid (Fig 6D)	Kruskal–Wallis	H=16.13	2	0.0002	Normality: p<0.05
Pyruvic acid (Fig. 6E)	Kruskal–Wallis	H=11.12	2	0.0009	Normality: p<0.05
Succinic acid (Fig. 6F)	Kruskal–Wallis	H=14.75	2	0.0001	Normality: p<0.05
		Lipid metabolism			
Arachidonic acid (Fig. 6G)	Kruskal–Wallis	H=13.66	2	< 0.0001	Normality: p<0.05
Dihomo-gamma-linolenic acid (Fig. 6H)	Kruskal–Wallis	H=11.47	2	0.0004	Normality: p<0.05
Eicosapentaenoic acid (Fig. 6I)	Kruskal–Wallis	H=14.00	2	< 0.0001	Normality: p<0.05

Note: Statistic: Represents the Kruskal–Wallis statistic (H). *df*: Degrees of Freedom. Assumption Checks: Data distribution was assessed using the Shapiro–Wilk test. Although individual metabolites may have shown normal distribution, the majority did not ($p < 0.05$). To maintain methodological consistency across the entire dataset, the non-parametric Kruskal–Wallis test was uniformly applied for all analyses. Significant differences between groups were determined using Dunn's multiple comparisons test with adjusted *p*-values.

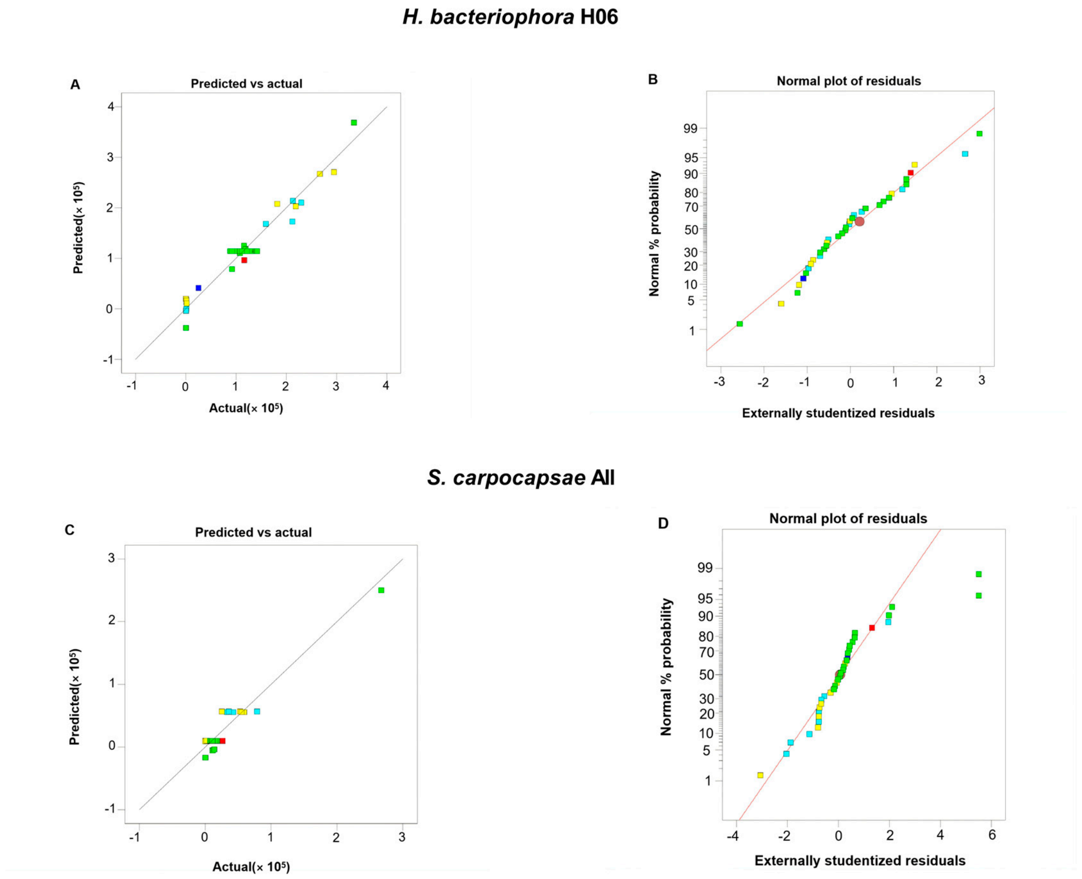

Supplementary Figure S1. Diagnostic plots for the Response Surface Methodology (RSM) models used in nematode yield optimization. **A–B** Model diagnostics for *H. bacteriophora* H06. **C–D** Model diagnostics for *S. carpocapsae* All. **A, C** Predicted vs. Actual values plots. The distribution of data points along the diagonal line demonstrates a high correlation between the experimental values (Actual) and the values predicted by the RSM model (Predicted), confirming the goodness of fit for both models ($R^2 >$ 0.9). **B, D** Normal probability plots of studentized residuals. The data points clustering closely around the straight line indicate that the residuals follow a normal distribution, confirming that the ANOVA assumptions were met.

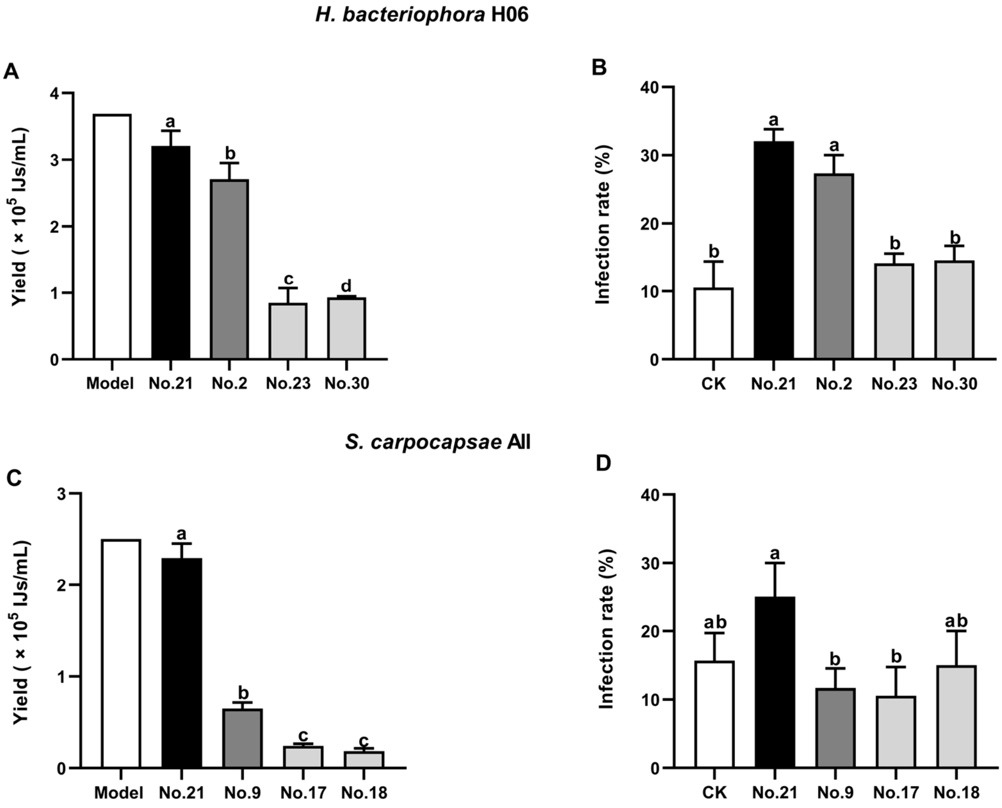

Supplementary Figure S2. Validation of yield and infectivity in a second independent experimental trial. To confirm the reproducibility of the "high-yield/high-quality" phenotype, the optimization validation (corresponding to Fig. 2) and infectivity assays (corresponding to Fig. 3A, D) were repeated in a temporally independent trial. **A, B** *Heterorhabditis bacteriophora* H06: **A** Yield validation, where the "Model" bar represents the theoretical yield predicted by RSM; **B** Infectivity against *G. mellonella* larvae. **C, D** *Steinernema carpocapsae* All: **C** Yield validation; **D** Infectivity against *G. mellonella* larvae. ***Note:*** Recovery and dispersal rates in this second trial also showed trends consistent with the primary trial presented in Fig. 3 (data not shown). Data are expressed as means ± SEM (n = 3 biological replicates). Different lowercase letters above bars indicate significant differences (one-way ANOVA followed by Tukey's HSD, $p < 0.05$). Statistical details: Yield: *H. bacteriophora* H06 (Panel A: $F_{3,8} = 107.4$, $p < 0.0001$); *S. carpocapsae* All (Panel C: $F_{3,8} = 389.5$, $p < 0.0001$). Infectivity: *H. bacteriophora* H06 (Panel B: $F_{4,10} = 41.36$, *p*

< 0.0001); *S. carpocapsae* All (Panel D: $F_{4,10} = 5.56$, $p = 0.0128$). For yield comparisons, the Model group (n = 1) was excluded from statistical analysis.

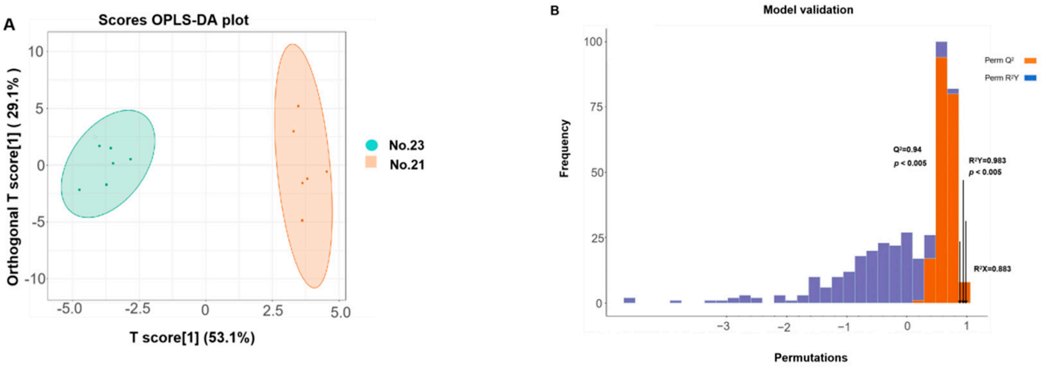

Supplementary Figure S3. Multivariate statistical analysis of the hydrophobic lipidomic profiles (free fatty acids) comparing the High Yield (No. 21) and Low Yield (No. 23) groups. **A** Orthogonal Partial Least Squares Discriminant Analysis (OPLS-DA) score plot. The clear separation along the predictive component (T score [1]) indicates distinct lipid metabolic signatures between the optimized high-yield group (No. 21) and the low-yield group (No. 23). Shaded ellipses represent the 95% confidence interval. **B** Permutation test (n = 200) for model validation. The validation plot confirms the robustness of the OPLS-DA model. The high values of explained variation ($R^2Y = 0.983$) and predictive capability ($Q^2 = 0.94$), along with a significant *p*-value ($p < 0.005$), confirm that the observed metabolic separation is statistically significant and not due to overfitting.

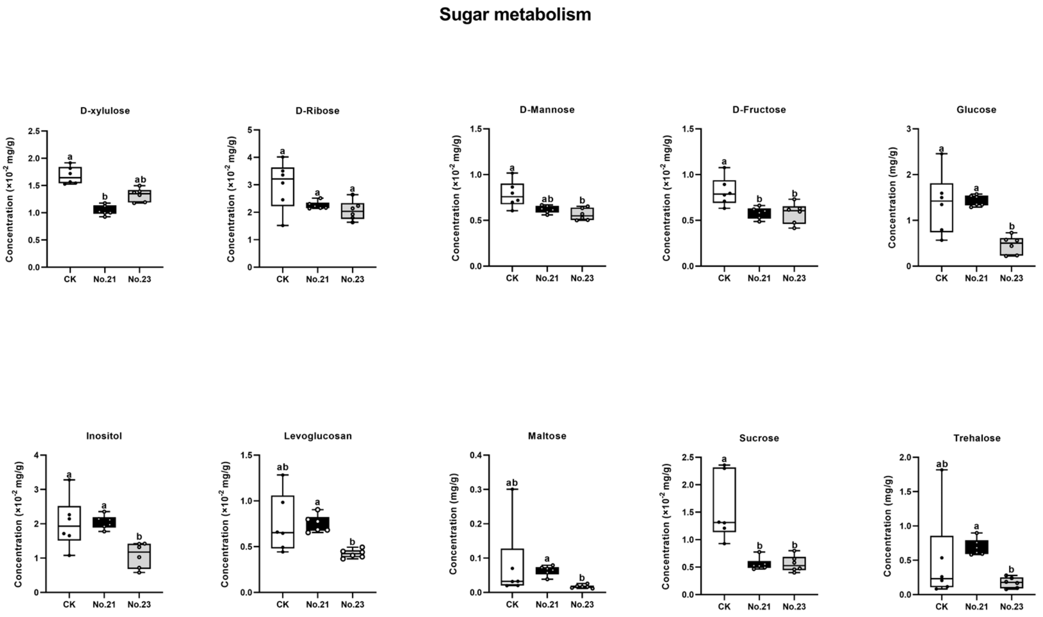

Supplementary Figure S4. Comprehensive metabolic profile of sugars and polyols in *Heterorhabditis bacteriophora* H06 infective juveniles. The panels display the relative abundance of detected carbohydrates and sugar alcohols across three culture conditions: solid culture control (CK), optimized high-yield liquid culture (No. 21), and low-yield liquid culture (No. 23). Data are presented as box plots overlaid with individual data points (n = 6). The central line indicates the median, and the box edges represent the interquartile range. Statistical significance was determined using the Kruskal–Wallis test followed by Dunn's multiple comparisons test. Different letters above the boxes indicate statistically significant differences ($p < 0.05$).

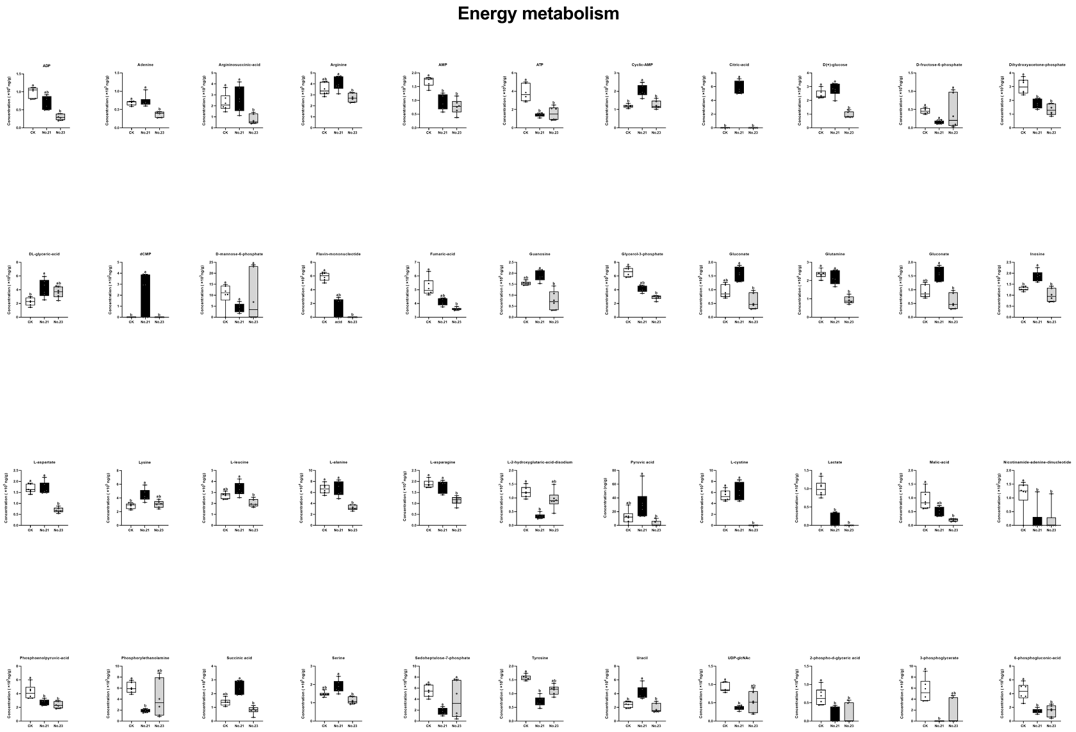

Supplementary Figure S5. Comparative analysis of TCA cycle intermediates and energy-related metabolites. This figure illustrates the variations in key organic acids involved in the tricarboxylic acid (TCA) cycle and central carbon metabolism. Comparison groups include the solid control (CK), optimized high-yield liquid group (No. 21), and low-yield liquid group (No. 23). The accumulation of specific intermediates (e.g., Succinic acid, Malic acid) in the optimized group indicates the reactivation of metabolic activity. Data are presented as box plots overlaid with individual data points (n = 6). The central line represents the median, the box limits indicate the interquartile range (IQR), and whiskers extend to the minimum and maximum values. Statistical differences were analyzed using the Kruskal–Wallis test followed by Dunn's post hoc test ($p < 0.05$).

Lipid metabolism

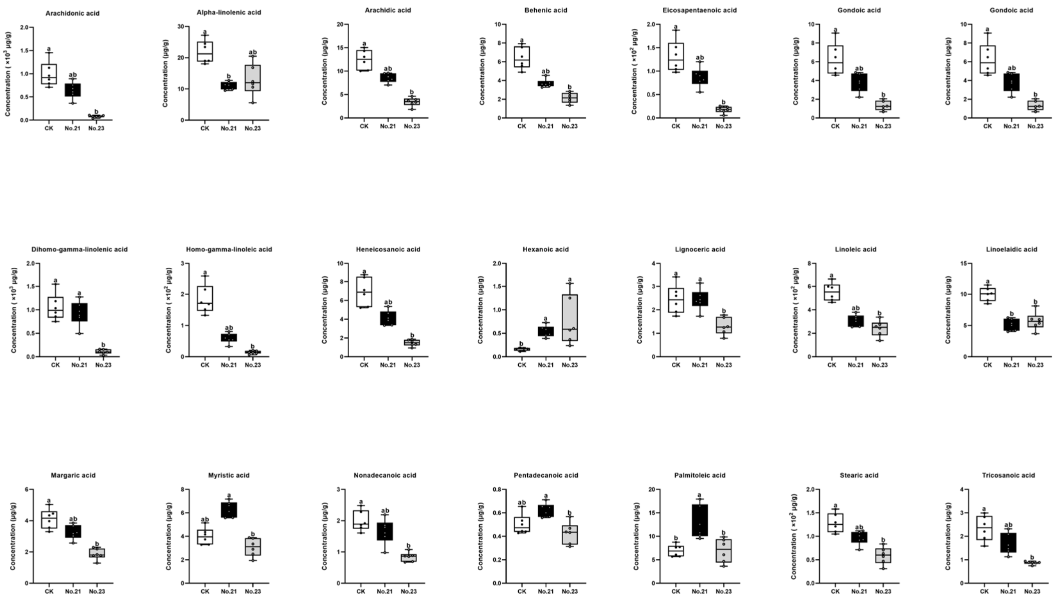

Supplementary Figure S6. Global profiling of free fatty acids (FFAs) and lipid composition. The panels show the quantitative changes in saturated and unsaturated fatty acids in *H. bacteriophora* IJs derived from different fermentation strategies. Note the specific accumulation of polyunsaturated fatty acids (PUFAs) such as EPA and ARA in the optimized liquid culture (No. 21) compared to the hypoxic low-yield group (No. 23). Data are presented as box plots with jitter points showing the distribution of six biological replicates. The box represents the median and interquartile range (IQR), while whiskers denote the range (min to max). Statistical analysis was performed using the Kruskal–Wallis test with Dunn's multiple comparison correction. Significant differences are denoted by distinct letters ($p < 0.05$).
